# CircFAT1 promotes hepatocellular carcinoma progression via miR‐30a‐5p/REEP3 pathway

**DOI:** 10.1111/jcmm.16085

**Published:** 2020-11-11

**Authors:** Hailiang Wei, Shuguang Yan, Yi Hui, Yonggang Liu, Hui Guo, Qian Li, Jingtao Li, Zhanjie Chang

**Affiliations:** ^1^ Department of General Surgery The Hospital Affiliated to Shaanxi University of Chinese Medicine Xianyang China; ^2^ College of Basic Medicine The Shaanxi University of Chinese Medicine Xianyang China; ^3^ Department of Liver Diseases The Hospital Affiliated to Shaanxi University of Chinese Medicine Xianyang China; ^4^ Medical Experiment Center The Shaanxi University of Chinese Medicine Xianyang China

**Keywords:** circFAT1, hepatocellular carcinoma, miR‐30a‐5p, REEP3

## Abstract

As newly found non‐coding RNAs, circular RNAs (circRNAs) are involved in multiple biological processes. Emerging evidence has illustrated the pivotal roles of circRNAs in various human cancers. However, the function of circFAT1 in hepatocellular carcinoma (HCC) remains largely unclear. In the present study, we found that circFAT1 expression is up‐regulated in HCC tissues and cells. In addition, circFAT1 level is positively correlated with TNM stage and tumour size. To further explore the function of circFAT1 in HCC, in vitro and in vivo experiments were performed. The results show that circFAT1 inhibition reduces the proliferation and invasion of HCC cells and tumorigenesis in vivo, whereas REEP3 overexpression reverses these processes. In conclusion, circFAT1 sponges miR‐30a‐5p to regulate the expression of REEP3, thus promoting hepatocarcinogenesis. New HCC diagnosis or treatment strategies may be developed from circFAT1 as a target.

## 1 INTRODUCTION

As the most common primary liver cancer, hepatocellular carcinoma (HCC) is the sixth leading cause of cancer‐related mortality worldwide, with an estimated 750 000 new deaths annually.^1^ Although therapeutic strategies of HCC have been improved greatly, HCC is still notorious for its poor prognosis, metastasis and recurrence.^2^ Therefore, novel therapeutic strategies and potential biomarkers focusing on the molecular mechanism of hepatocarcinogenesis are of great significance.

Widely found in eukaryotes, circular RNAs (circRNAs) are a subclass of endogenous non‐coding RNAs bearing a closed covalent loop.^3^ It has been proved that circRNAs are mainly conserved in the cytoplasm, and they are stable and not easily degraded by RNA exonuclease.^4^ In recent years, increasing studies have shown that circRNAs participate in various biological processes, including acting as miRNA sponge, and regulating protein binding and gene transcription.^5^, ^6^ Notably, the role of circRNAs in multiple cancers may show their potential as diagnostic biomarkers.^7^ For example, circCCDC9 was down‐regulated in gastric cancer and acted as the sponge of miR‐6792‐3p to regulate the expression of CAV1, thus suppressing the progression of gastric cancer^8^; circTADA2A promoted the progression and metastasis via miR‐203a‐3p/CREB3 axis.^9^ However, the role of circRNAs in HCC progression remains obscure.

MicroRNAs (miRNAs), a class of small conservative non‐coding RNAs about 20‐24 nucleotides in length, can repress the expression of target gene by binding to the 3′UTR of mRNAs.^10^ Studies have reported that miRNAs are involved in multiple biological processes, such as the proliferation, invasion and apoptosis of cancer cells.^11^, ^12^ In addition, it is recognized that circRNAs act as a miRNA sponge to control cancer progression.^13^ For instance, circRNA_100290 sponged miR‐29 to facilitate the progression of oral squamous cell carcinomas.^14^ However, the knowledge of circRNA‐miRNA network in HCC progression is still porous.

In this study, we found that circFAT1 expression was significantly up‐regulated in HCC cells and tissues. CircFAT1 enhanced the expression of *REEP3* by acting as miR‐30a‐5p sponge and ultimately promoted the development of HCC. Our results presented a new potential diagnostic and therapeutic biomarker of hepatocellular carcinoma.

## 2 MATERIALS AND METHODS

### 2.1 Tissue samples and cell lines

The fresh‐frozen HCC tissues were obtained from patients at the Hospital Affiliated to Shaanxi University of Chinese Medicine. This study was approved by the Ethics Committee of the Hospital Affiliated to Shaanxi University of Chinese Medicine. The consent of each patient was obtained before the study started.

293T cells and HCC cell lines were obtained from The Cell Bank of Type Culture Collection of the Chinese Academy of Sciences (Shanghai, China). All cell lines were cultured in Dulbecco's modified Eagle's medium (DMEM) supplemented with 10% foetal bovine serum (FBS), 100 U/mL penicillin and 100 μg/mL streptomycin under 5% CO_2_ at 37°C.

### 2.2 RNA extraction and qRT‐PCR

Total RNA of HCC tissues and cell lines was extracted using TRIzol reagent (Invitrogen) as recommended by the manufacturer, and NanoDrop 2000 (Thermo Fisher Scientific) was used to measure the concentration and purity of RNA. The qualified RNA was reverse transcribed to cDNA using PrimeScript RT Master Mix (Takara). For circRNA and mRNA, the qRT‐PCR was performed using SYBR Premix Ex Taq (Takara) and the reactions were run on the ABI 7500 Real‐Time PCR System (Life Technologies). GAPDH was used as internal control. For miRNA, SYBR PrimeScript miRNA RT‐PCR Kit (Takara) was used for qRT‐PCR, and U6 was applied as an internal control. The relative expression levels of circRNA, miRNA and mRNA were calculated with 2^−ΔΔCT^ algorithm. Experiments were repeated for three times, each with three replicates.

### 2.3 RNase R treatment

Total RNA (10 μg) from Huh7 cells was incubated with 3 U/μg RNase R (Epicentre Technologies) for 15 minutes at 37°C. Subsequently, the treated RNA was reverse transcribed and verified by qRT‐PCR assay.

### 2.4 Fluorescence in situ hybridization (FISH)

FISH assay was used to detect the location of circFAT1 in HCC cells. Alexa Fluor 488‐labelled circFAT1 probes were synthesized by RiboBio, and hybridization was performed with the specific probes. The images were captured by a fluorescence microscope (Olympus).

### 2.5 Cell transfection

The sense sequence of sh‐circFAT1 (GAGAAAGATTCCCGACAGTTA) and antisense sequence were inserted into the pBLOCK‐iT 6‐DEST vectors (Thermo) to construct the sh‐circFAT1 plasmids. miR‐30a‐5p mimics (3′‐GAAAGGUCAGCUCCUACAAAUGU‐5′) and miR‐30a‐5p inhibitors (3′‐CTTCCUGTCGUGGUTGTTTUCU‐5′) were purchased from RiboBio. The OE‐circFAT1 and OE‐REEP3 plasmids were constructed using pLO‐ciR vectors (Geneseed) and pcDNA3.1 vectors, respectively. All plasmids transfected into Huh7 and Hep3B cells by using Lipofectamine 3000 (Invitrogen).

### 2.6 Luciferase reporter assay

The pGL3 Luciferase Reporter Vectors (Promega) containing the circFAT1‐MT, circFAT1‐MuT, *REEP3*‐WT or *REEP3*‐WuT sequences were co‐transfected with miR‐30a‐5p mimic or NC‐mimic into 293T and Huh7 cells. After 24 hours of incubation, 1× PLB was used to lyse the cells, and the luciferase activities were measured using the Dual‐Luciferase Reporter Assay System (Promega).

### 2.7 RNA immunoprecipitation (RIP)

RIP assay was performed by using Magna RIP™ RNA‐Binding Protein Immunoprecipitation Kit (Millipore) according to the manufacturer's instructions. Huh7 and Hep3B cells were lysed in the RIP Lysis Buffer and incubated 6 hours at 4°C with the magnetic beads conjugated with anti‐Argonaute 2 (AGO2) or anti‐IgG antibody. Then, RNAs were extracted, and the purified RNAs were analysed by qRT‐PCR assay.

### 2.8 Cell counting kit‐8 proliferation assay

3 × 10^3^ Huh7 and Hep3B cells were seeded into 96‐well plates, and 10 μL of CCK‐8 reagent (Dojindo Corp) was added into the culture medium at the indicated time‐points. After 2 hours of incubation, the optional density (OD) at 450 nm was measured by the microplate reader (Thermo). Experiments were repeated for three times.

### 2.9 Transwell invasion assay

The Transwell chambers (Corning) were paved with Matrigel mix for invasion assays. HCC cells suspended in the medium without foetal bovine serum were seeded into the upper chamber. Medium containing 10% foetal bovine serum was infused into the bottom chamber. After 24 hours, the upper chamber was fixed by 4% paraformaldehyde. The cells with crystal violet for 15 minutes. Experiments were repeated for three times.

### 2.10 Western blot

HCC cells were lysed using RIPA buffer (Sangon). After measuring protein concentration, 30 μg of total protein was separated by 10% sodium dodecyl sulphate‐polyacrylamide gel electrophoresis (SDS‐PAGE) and transferred to polyvinylidene fluoride (PVDF) membranes (Millipore). 5% skimmed milk was used to block the membranes at room temperature for 2 hours. Then, the membranes were incubated with the primary antibody (1:10 000) overnight at 4°C. The membranes were washed for three times with phosphate‐buffered solution containing 0.5% Tween‐20 (PBST) and incubated with the HRP‐conjugated secondary antibody (1:10 000) at room temperature for 1 hour. Subsequently, the protein bands were visualized using the Clarity Western ECL Substrate (Bio‐Rad).

### 2.11 Xenografts in mice

4‐week‐old female athymic BALB/c nude mice were purchased from Shanghai Laboratory Animal Center (SLAC). 1 × 107 Huh7 cells stably transfected with sh‐circFAT1 plasmids or controls were subcutaneously injected into the mice. At 21 days after injection, the tumour tissues were excised and measured.

### 2.12 Statistical analyses

Statistical analyses were performed using GraphPad Prism 7 and SPSS 22.0 (IBM, Chicago, USA). Student's *t* test and one‐way analysis of variance were used to analyse the difference between groups. *P* < .05 was considered statistically significant. Each experiment has three independent replicates.

## 3 RESULTS

### 3.1 The expression of circFAT1 is up‐regulated in HCC tissues and cell lines and is predominantly located in the cytoplasm

**FIGURE 1:**
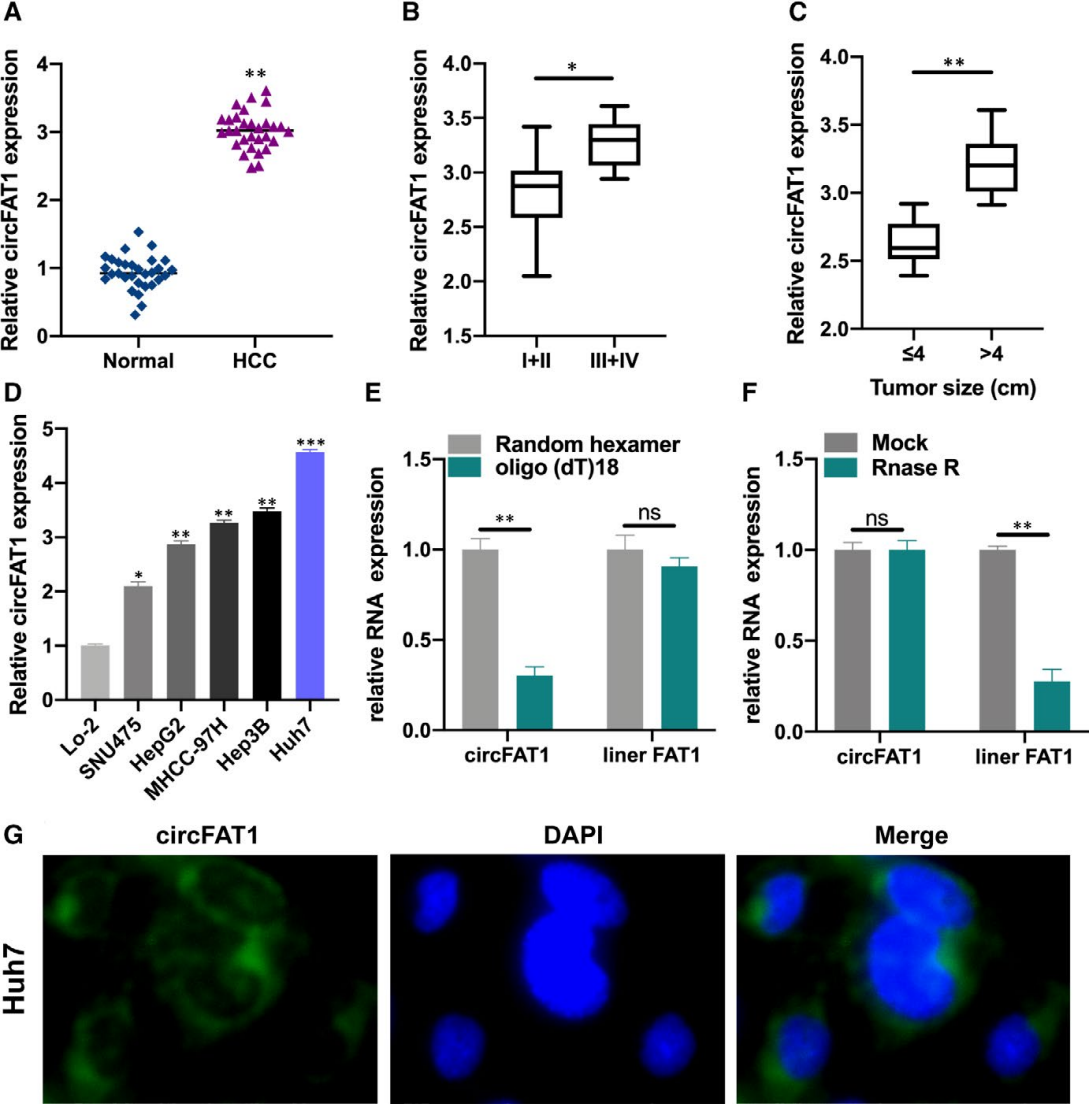
The expression of circFAT1 is up‐regulated in HCC tissues. (A) Relative expression of circFAT1 in 30 pairs of HCC tissues and its normal matched tissues was measured by qRT‐PCR. (B) Relative expression of circFAT1 in different HCC TNM stages was detected by qRT‐PCR. (C) Expression level of circFAT1 in HCC tumour samples with different tumour size was analysed by qRT‐PCR. (D) qRT‐PCR was conducted to measure the expression of circFAT1 in different HCC cell lines and normal liver cell Lo‐2. (E) Relative expression of circFAT1 and liner RNA FAT1 in Huh7 cells was analysed by qRT‐PCR. (F) Relative expression of circFAT1 and liner RNA FAT1 in Mock or RNase R‐treated Huh7 cells. (G) FISH assays were performed to detect the location of circFAT1 in Huh7 cells. Scale bars: 100μm. All experiments were repeated at least three times. *P < .05, **P < .01, ***P < .001; ns stands for no significance

To explore the correlation between circFAT1 expression and HCC development, we collected 30 pairs of HCC tissues and normal tissues, and detected circFAT1 expression by qRT‐PCR (Figure 1A). The expression of circFAT1 in HCC tissue samples was significantly increased. In addition, the circFAT1 expression in HCC samples of different TNM stage or size was also analysed by qRT‐PCR (Figure 1B,C). Obviously, the circFAT1 expression in III+IV TNM stage or large size tumours was higher. Consistently, the expression of circFAT1 in HCC cell lines was much higher than in normal liver cells (Figure 1D), and the highest in Huh7 and Hep3B cell lines. Thus, Huh7 and Hep3B cell lines were selected to investigate the regulatory mechanism of circFAT1 in HCC. To verify the circular feature of circFAT1, we used random hexamer and oligo(dT)18 to amplify circFAT1 and liner FAT1, respectively (Figure 1E). The results proved that circFAT1 was a circular RNA. RNase R treatment also confirmed the circular structure of circFAT1 (Figure 1F). FISH assays displayed that circFAT1 was mainly localized in the cytoplasm (Figure 1G).

### 3.2 Down‐regulating circFAT1 inhibits HCC cell proliferation, invasion and EMT

**FIGURE 2:**
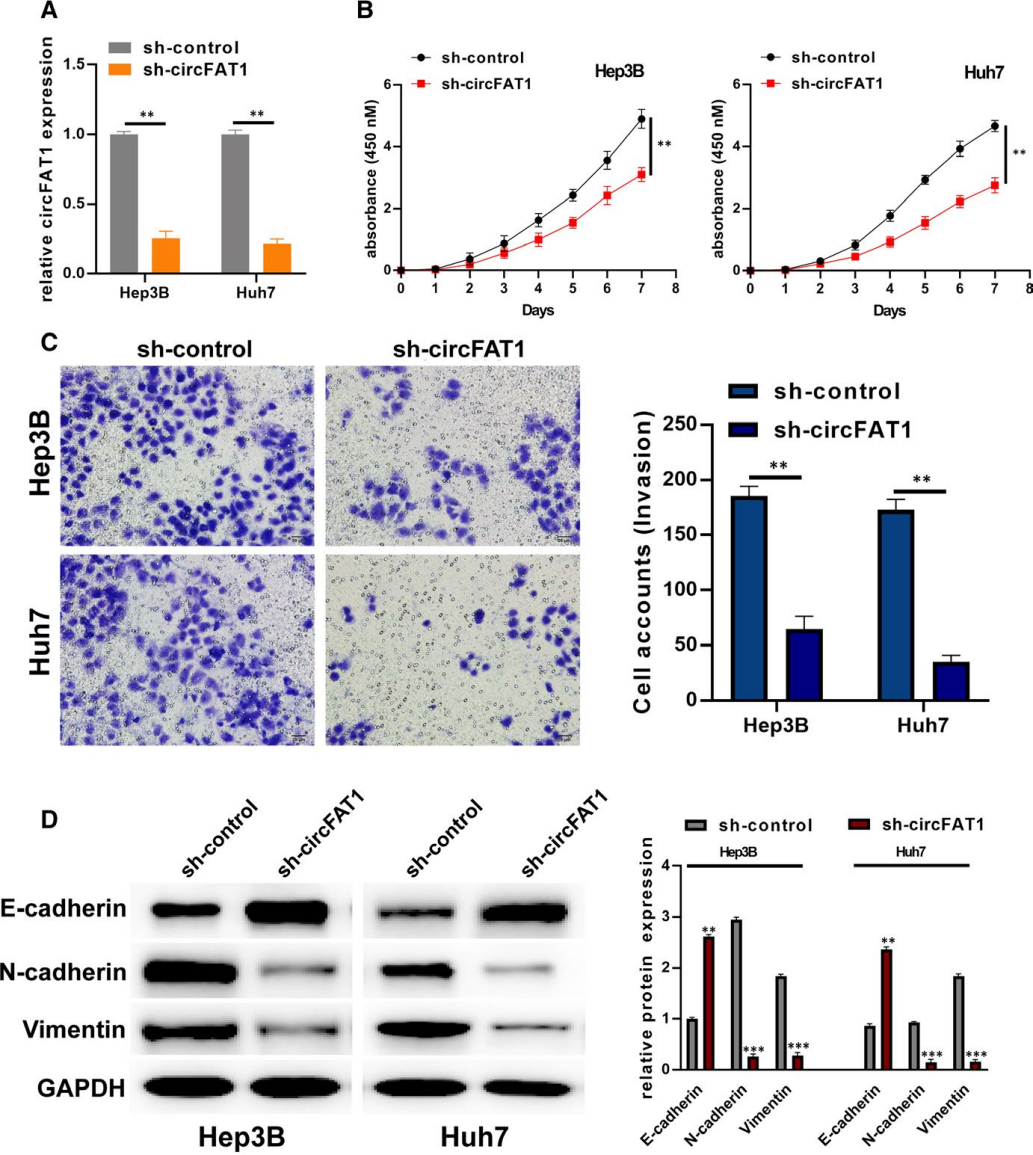
Down‐regulated circFAT1 inhibits HCC cell proliferation, invasion and EMT level. (A) Hep3B and Huh7 cells were, respectively, transfected with sh‐control and sh‐circFAT1. The transfection efficiencies were analysed by qRT‐PCR. (B) Determination of shRNA‐treated Hep3B and Huh7 cells proliferation by performing CCK‐8 assays. (C) Cell invasion was detected by using transwell invasion assays. Invasion cells were amounted and shown. (D) The expression of EMT protein biomarkers E‐cadherin, N‐cadherin and vimentin in shRNA‐treated Hep3B and Huh7 cells was measured by Western blot; the expression of EMT proteins was analysed. All experiments were repeated at least three times.**P < .01,***P < .001

The small hairpin RNAs (shRNAs) of circFAT1, which could stably knock down circFAT1 expression in HCC cell lines, were designed and cloned into the corresponding vectors. Vectors carrying sh‐circFAT1 and negative controls were transfected into Hep3B and Huh7 cells, respectively. The expression of circFAT1 in the transfected cells was measured by qRT‐PCR. As shown in Figure 2A, circFAT1 was remarkably silenced. Subsequently, CCK‐8 assay showed that the down‐regulation of circFAT1 significantly inhibited the cell proliferation, indicating that circFAT1 has a crucial effect on maintaining a high proliferation rate (Figure 2B). Moreover, Transwell assay found that circFAT1 shRNA prominently reduced the invasion of HCC cells (Figure 2C).

Then, the expression of EMT biomarkers E‐cadherin, N‐cadherin and vimentin in shRNA‐treated Hep3B and Huh7 cells was measured. As the results show, when circFAT1 was knocked down, the expression of E‐cadherin was increased, but N‐cadherin expression and vimentin expression were significantly reduced (Figure 2D). The above results illustrate that down‐regulating circFAT1 can inhibit HCC cell proliferation, invasion and EMT.

### 3.3 CircFAT1 acts as miR‐30a‐5p sponge

**FIGURE 3:**
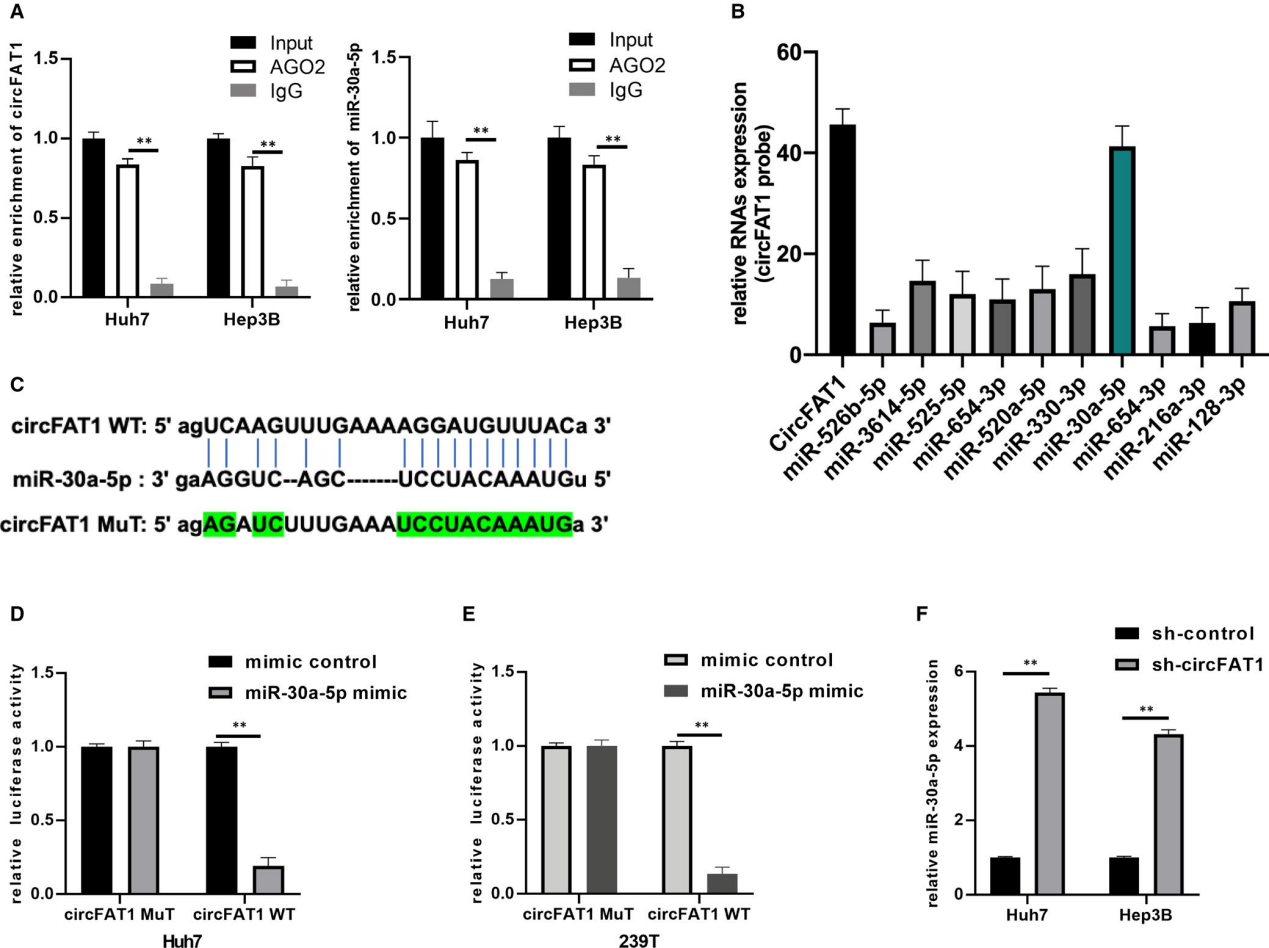
CircFAT1 acts as miR‐30a‐5p sponge. (A) RIP experiments with AGO2 antibody were conducted, and qRT‐PCR assays were used to detect the expression of circFAT1 and miR‐30a‐5p. (B) qRT‐PCR assays with specific circFAT1 probe were performed to measure the expression of potential sponging RNAs (Starbase 3.0). (C) The putative binding sites between circFAT1 WT/ MuT and miR‐30a‐5p. (D and E) Luciferase activity was detected in luciferase reporter vectors harbouring circFAT1 WT/MuT sequences and miR‐30a‐5p mimics co‐transfected Huh7 and 293T cells. (F) Determination of the miR‐30a‐5p in sh‐NC or sh‐circFAT1 transfected Huh7 and Hep3B cells. All experiments were repeated at least three times. **P < .01

Previous research has reported that circRNAs can act as miRNA sponges to repress their activity. To investigate whether circFAT1 regulates the biological processes in HCC via sponging miRNAs, we conducted RIP experiment with AGO2 antibody, and qRT‐PCR assay was used to detect the expression of circFAT1. As shown in Figure 3A, the endogenous circFAT1 in Hep3B and Huh7 cells was more enriched in AGO2 group than in IgG group, suggesting that circFAT1 can bind to miRNAs through AGO2 protein. Then, the potential target miRNAs were predicted by Starbase 3.0. qRT‐PCR assay using specific circFAT1 probes was performed to measure the expression of potential miRNAs. Among them, miR‐30a‐5p was the most possible target (Figure 3B). To validate this finding, dual‐luciferase reporter assay was applied. The luciferase reporter vectors carrying the sequences of circFAT1‐WT and circFAT1‐MuT were constructed, respectively. Then, miR‐30a‐5p mimics/mimic controls and vectors harbouring circFAT1 WT/MuT were co‐transfected into Huh7 and 293T cells. The results showed that miR‐30a‐5p overexpression inhibited the luciferase activity in circFAT1 WT group, but brought no changes in circFAT1 MuT group (Figure 3D,E). The expression of miR‐30a‐5p in Huh7 and Hep3B cells transfected with sh‐control or sh‐circFAT1 was determined by qRT‐PCR (Figure 3F). It proved that circFAT1 overexpression decreased miR‐30a‐5p expression. To sum up, there is a direct interaction between circFAT1 and miR‐30a‐5p, and circFAT1 acts as miR‐30a‐5p sponge.

### 3.4 MiR‐30a‐5p directly interacts with *REEP3*

**FIGURE 4:**
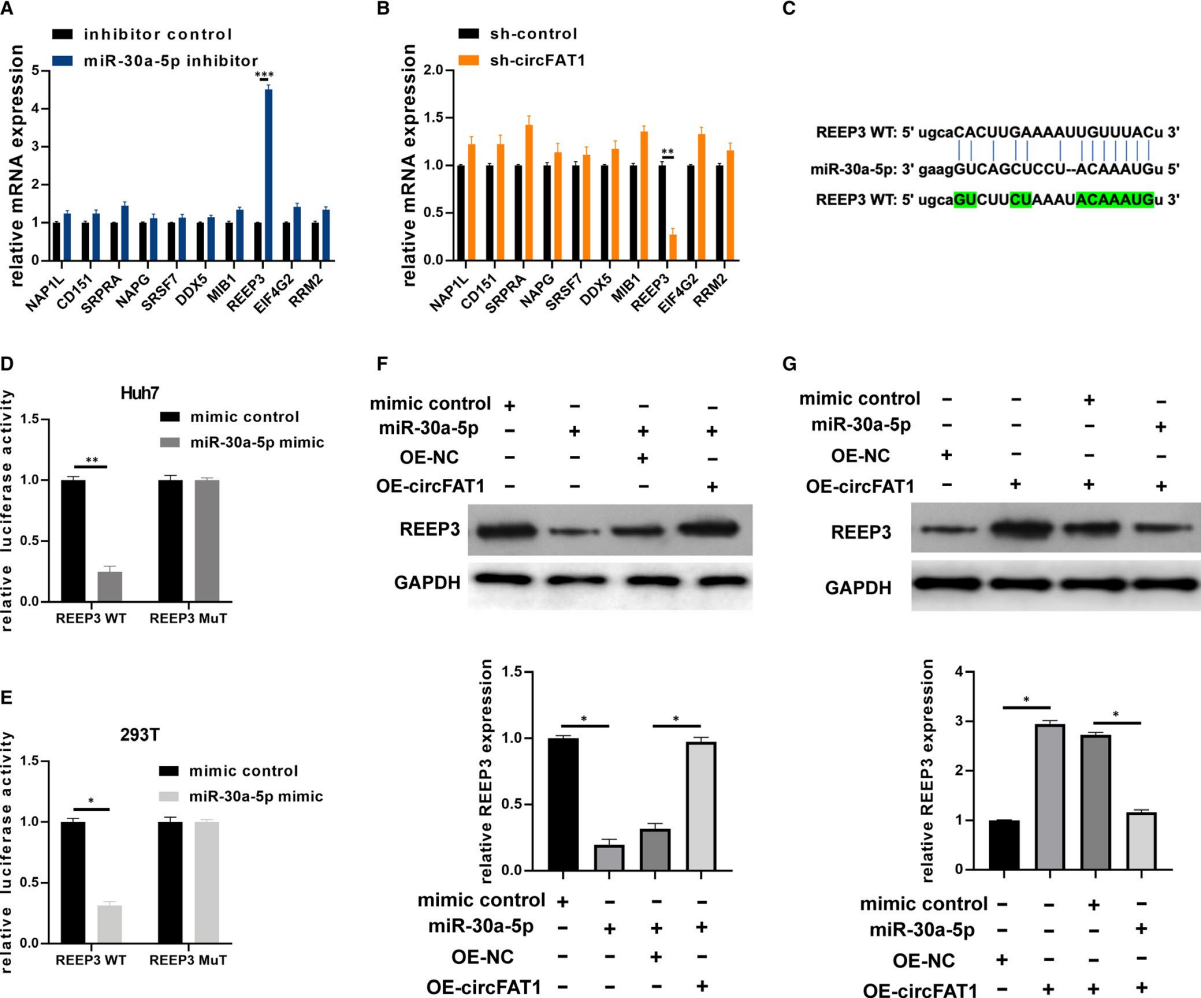
MiR‐30a‐5p directly interacts with REEP3. (A) Determination of selected mRNAs expression by qRT‐PCR in Huh7 cells transfected with inhibitor control or miR‐30a‐5p inhibitor, respectively. (B) Measurement of selected mRNAs expression using qRT‐PCR in Huh7 cells transfected with sh‐control or sh‐circFAT1, respectively. (C) The putative binding sites between REEP3 wild‐type (WT) or mutant type (MuT) and miR‐30a‐5p. (D and E) Luciferase activity was detected in luciferase reporter vectors harbouring REEP3 WT or Mut sequences and miR‐30a‐5p mimics co‐transfected Huh7 and 293T cells. (F) Huh7 cells were pre‐transfected with mimic controls, miR‐30‐5p mimics, miR‐30‐5p mimics + OE‐NC and miR‐30‐5p mimics + OE‐circFAT1, respectively. The expression of REEP3 in treated Huh7 cells was detected by Western blot. (G) Huh7 cells were pre‐transfected with OE‐NC, OE‐circFAT1, NC‐mimic + OE‐circFAT1 and miR‐30‐5p mimic + OE‐circFAT1, respectively. The expression of REEP3 in treated Huh7 cells was detected by Western blot. Comparative statistics were shown. All experiments were repeated at least three times. *P < .05, **P < .01, ***P < .001

To clarify which target gene is regulated by miR‐30a‐5p in HCC, we screened out the differentially expressed genes in Huh7 cells transfected with miR‐30a‐5p inhibitor and the control Huh7 cells. QRT‐PCR assay was used to detect the mRNA expression of 10 selected genes. Silencing miR‐30a‐5p significantly decreased the mRNA expression of *REEP3* in Huh7 cell line (Figure 4A). We next measured the mRNA expression of the 10 genes in Huh7 cells transfected with sh‐control or sh‐circFAT1. As shown in Figure 4B, circFAT1 expression had a significant positive correlation with *REEP3* expression, which is a characteristic of competing endogenous RNA (ceRNA). The putative binding sites between miR‐30a‐5p and *REEP3* WT or MuT were shown in Figure 4C. To verify the interaction between miR‐30a‐5p and *REEP3*, luciferase assay was performed. Luciferase reporter vectors carrying *REEP3* WT/MuT sequences and miR‐30a‐5p mimics/ mimic controls were co‐transfected into Huh7 and 293T cells. Luciferase activity was reduced when miR‐30a‐5p expression was up‐regulated in group *REEP3* WT, but no suppression was observed in group *REEP3* MuT, indicating that miR‐30a‐5p can directly bind to *REEP3* (Figure 4D,E). Furthermore, the protein expression of *REEP3* in Huh7 cells transfected with mimic controls, miR‐30‐5p mimics, miR‐30‐5p mimics + OE‐NC and miR‐30‐5p mimics + OE‐circFAT1 was detected by Western blot. The results showed that miR‐30a‐5p overexpression inhibited the protein expression of *REEP3*, a process that can be reversed by circFAT1 overexpression (Figure 4F). Additionally, circFAT1 overexpression increased the protein expression of *REEP3*, but in the group co‐transfected with OE‐circFAT1 and miR‐30a‐5p mimics, the *REEP3* expression was decreased again (Figure 4G). The above results illustrate that circFAT1 can regulate the expression of *REEP3* through sponging miR‐30a‐5p.

### 3.5 CircFAT1 promotes HCC development through miR‐30a‐5p/*REEP3* pathway

**FIGURE 5:**
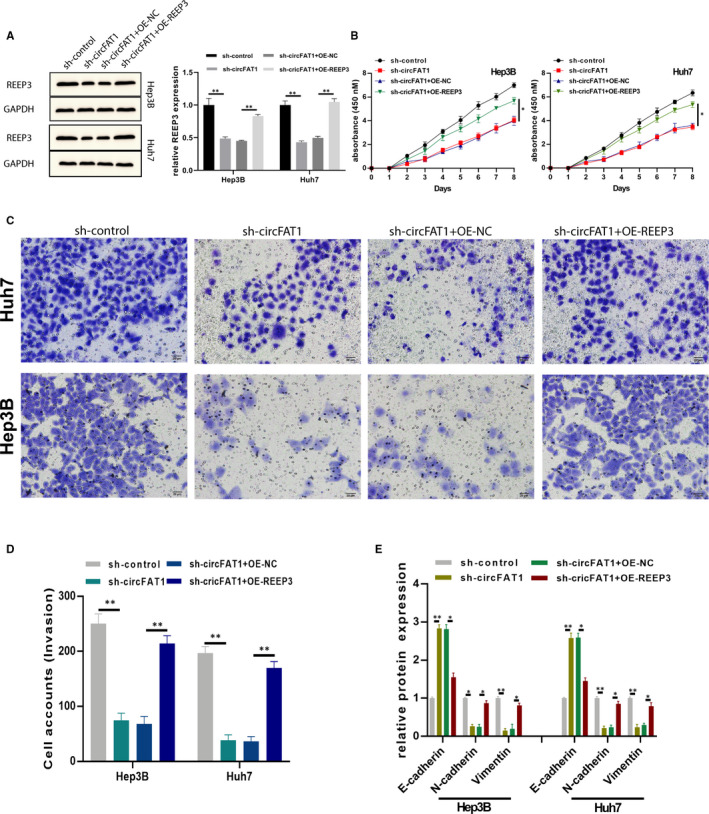
CircFAT1 promotes HCC development through miR‐30a‐5p/REEP3 pathway. HCC cell lines Hep3B and Huh7 were pre‐transfected with sh‐control, sh‐circFAT1, sh‐circFAT1 + OE‐NC and sh‐circFAT1 + OE‐REEP3, respectively. (A) The expression of REEP3 was detected using Western blot. (B) To detect cells proliferation ability, treated Hep3B and Huh7 cells were subjected to CCK‐8 assays. (C‐D) Transwell invasion assays were conducted to detect cells invasion ability, invasion cells were counted, and comparative statistics were shown. (E) The EMT biomarkers E‐cadherin, N‐cadherin and Vimentin level in treated cells were measured by ELISA assays. All experiments were repeated at least three times. *P < .05, **P < .01

To further investigate whether circFAT1 promotes the HCC development by targeting *REEP3*, *REEP3* overexpression plasmids were constructed and transfected into circFAT1‐silenced Huh7 and Hep3B cells. As shown in Figure 5A, *REEP3* expression was decreased by sh‐circFAT1 and OE‐REEP3 plasmids restored *REEP3* expression. CCK‐8 assay showed that *REEP3* overexpression reversed the inhibitory effect induced by circFAT1 down‐regulation in Huh7 and Hep3B cells (Figure 5B). The invasion was also rescued by *REEP3* (Figure 5C,D). Additionally, the EMT biomarkers E‐cadherin, N‐cadherin and vimentin expressions in treated cells were measured by ELISAs. As shown in Figure 5E, the obvious expressional changes confirmed the crucial role of circFAT1 and *REEP3* in regulating EMT (Figure 5E). Collectively, these findings reveal that circFAT1 promotes the proliferation, invasion and EMT of HCC cells by targeting *REEP3*.

### 3.6 CircFAT1 knockdown inhibits the tumorigenesis of HCC in vivo

**FIGURE 6:**
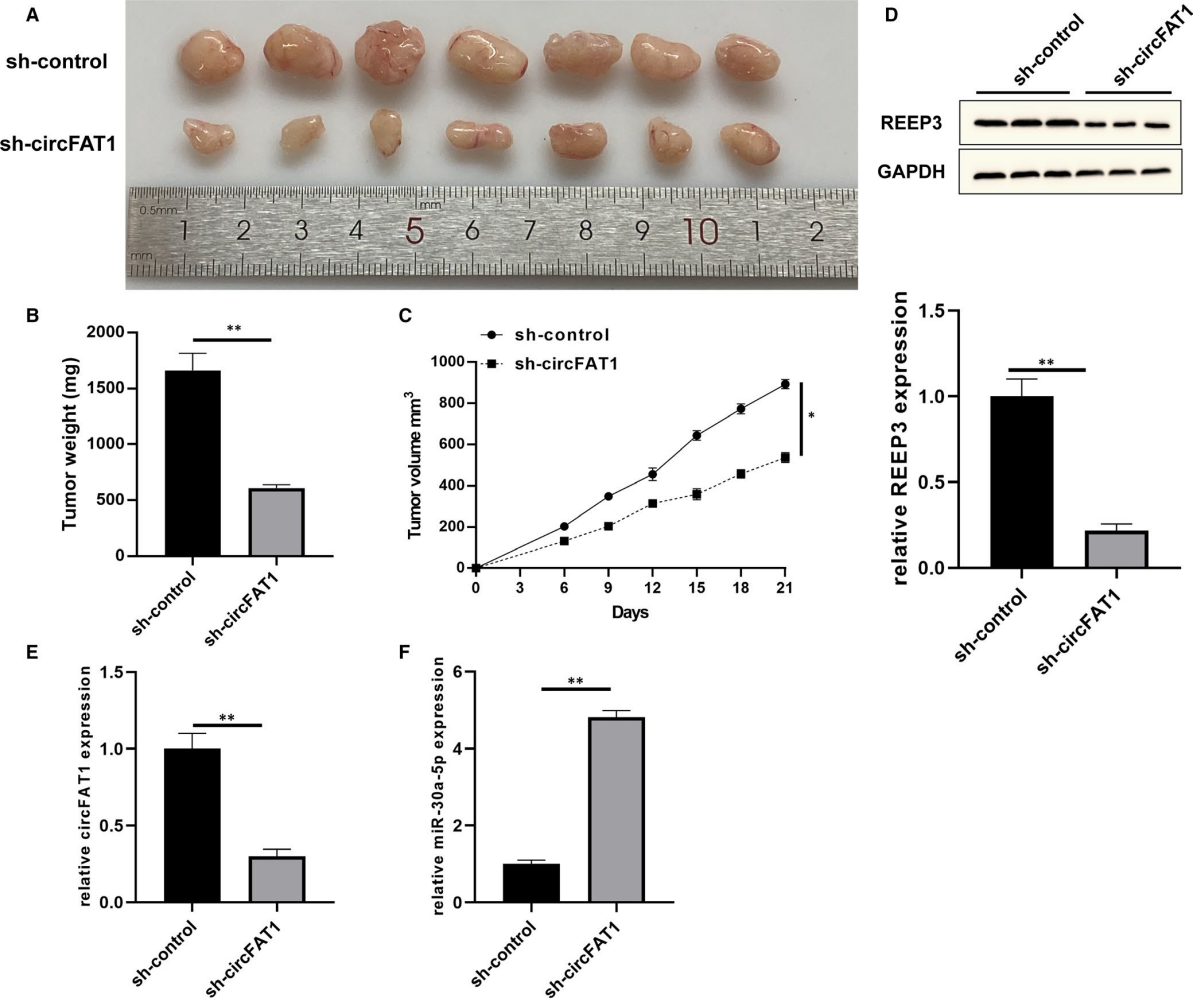
CircFAT1 knockdown inhibits tumorigenesis of HCC in vivo. Nude mice were subcutaneously injected with Huh7 pre‐transfecting with sh‐control or sh‐circFAT1 (n = 7 for each group). (A) The presentative image of xenograft tumours was shown. (B) Tumour weights were recorded, and the comparative statics were shown. (C) Tumour volumes were recorded 3 d a time, since day 6. (D‐F) The REEP3, circFAT1 and miR‐30a‐5p expression in xenograft tumours. All experiments were repeated at least three times. **P < .01

To further explore the function of circFAT1 in vivo, a xenograft tumour model was established through subcutaneously injecting Huh7 cells stably transfected with sh‐circFAT1 and sh‐control into BALB/c nude mice. The tumours are shown in Figure 6A. Tumour weights are shown in Figure 6B, and tumour volumes were recorded every 3 days from day 6 after injection (Figure 6C). Compared with the sh‐control group, the tumour weights and volumes in sh‐circFAT1 group were reduced significantly. In addition, we determined the *REEP3* (Figure 6D), circFAT1 (Figure 6E) and miR‐30a‐5p (Figure 6F) expression in these tumours. The results suggest that circFAT1 knockdown suppresses the growth of HCC in vivo by regulating miR‐30a‐5p and *REEP3*.

## 4 DISCUSSION

In recent years, increasing studies have shown that circRNAs play an important role in multiple biological processes, especially the development of cancers.^15^ According to reports, circRNAs may function as tumour promoters or suppressors in various cancers, such as gastric cancer, lung cancer and bladder cancer.^16^, ^17^, ^18^ However, just few circRNAs have been recognized to work in HCC. Our study is the first to reveal the mechanism of circFAT1 in HCC development.

Cancer‐related studies are focusing on the regulatory role of ‘circRNA‐miRNA‐mRNA’ axis.^19^, ^20^ Many circRNAs contain miRNAs response elements and act as miRNA sponges in the cytoplasm.^21^ For example, has_circ_001680 promotes the progression of CRC through miR‐340/BMI1 pathway^22^; has_circ_100395 inhibits the proliferation, migration and invasion of lung cancer cells through modulating miR‐1228/TCF21 axis.^23^ In our study, we found that circFAT1 was significantly up‐regulated in HCC tissues and inversely correlated with HCC TNM stage and tumour size. Through in vivo and in vitro functional experiments, we verified that circFAT1 promoted the proliferation, invasion and EMT of HCC cells. In addition, our RIP experiment and the luciferase reporter assay confirmed that circFAT1 was localized in the cytoplasm and directly bound to miR‐30a‐5p, a target predicted by bioinformatics analysis. Subsequently, we discovered that *REEP3* was the target gene of miR‐30a‐5p, a gene located at human genome 10q21.3.^24^ Our research is the first to confirm that circFAT1 can promote the progression of HCC by sponging miR‐30a‐5p and enhancing *REEP3*.

However, there are still several limitations to this study. First, we tested the level of circFAT1 in HCC tissues and cells, but whether it can be detected in body fluids still needs further study. In addition, circFAT1 might bind to other miRNAs. Therefore, the potential of circFAT1 in HCC diagnosis and treatment needs to be well described in further investigation. In conclusion, our data indicate that circFAT1 promotes the progression of HCC by sponging miR‐30a‐5p to regulate *REEP3* expression. New HCC diagnosis or treatment strategies may be developed from circFAT1 as a target.

## CONFLICT OF INTEREST

The authors confirm that there are no conflicts of interest.

## AUTHOR CONTRIBUTIONS

**Hailiang Wei:** Investigation (lead); methodology (equal); project administration (lead); writing‐original draft (lead). **Shuguang Yan:** Investigation (supporting); methodology (equal); project administration (supporting); writing‐original draft (supporting). **Yi Hui:** Investigation (supporting); methodology (supporting); visualization (equal). **Yonggang Liu:** Methodology (supporting); visualization (supporting). **Hui Guo:** Methodology (supporting); visualization (supporting). **Qian Li:** Methodology (supporting); visualization (supporting). **Jingtao Li:** Conceptualization (equal); funding acquisition (equal); writing‐review & editing (equal). **Zhanjie Chang:** Funding acquisition (equal); project administration (equal); writing‐review & editing (equal).

## Data Availability

The data that support the findings of this study are available from the corresponding author upon reasonable request.
